# EV-derived small non-coding RNAs from porcine follicular fluid regulate follicle development using Pandora sequence

**DOI:** 10.3389/fvets.2026.1754433

**Published:** 2026-03-17

**Authors:** Junhe Hu, Shuting Peng, Wei Deng, Zhenzhen Guo, Juan Wu, Xiansheng Tan, Zhi Zeng, Aiqun Wang

**Affiliations:** 1 College of Agriculture and Biotechnology, Hunan University of Humanities, Science and Technology, Loudi, Hunan, China; 2 Shuangfeng County Yiqun Cultivation and Breeding Farmers' Professional Cooperative, Loudi, Hunan, China

**Keywords:** extracellular vesicles (EVs), follicular development, Pandora sequence, porcine follicular fluid (PFF), small non-coding RNAs (SncRNAs)

## Abstract

**Background:**

Follicular fluid extracellular vesicles (EVs) have emerged as critical mediators of intercellular communication during oocyte development. This study investigates the role of small non-coding RNAs (SncRNAs) within porcine follicular fluid extracellular vesicles in regulating oocyte maturation, with a focus on functional disparities between small (group S: < 3 mm) and large (group L:>6 mm) follicles.

**Methods:**

Extracellular vesicles were isolated from porcine follicular fluid via ultracentrifugation and characterized by nanoparticle tracking analysis (NTA), transmission electron microscopy (TEM), and nanoflow cytometry for CD63 and CD81 positive expression. Pandora sequencing of SncRNAs identified differentially expressed SncRNAs (piRNAs, rRNAs, tsRNAs, miRNAs, and snoRNAs) across follicle sizes using Pandora sequence.

**Results:**

Firstly, SncRNA composition analysis demonstrated distinct proportional distributions: PiRNA constituted 43% in group L vs. 51.8% in S follicles, while tsRNA showed an inverse trend (25.3% in group L vs. 14.2% in group S). Subtype-specific differences were prominent in tsRNAs, with 3′ tsRNA accounting for 7.8% in group S follicles vs. 0.3% in group L, and 5′ tsRNA representing 13.8% in S vs. 9.5% in group L. Secondly, differential expression analysis identified eight highly upregulated SncRNAs in group L follicles, including upregulated piR-1060463, piR-1205752, miR-10b, miR-29b, miR-100, miR-221-3p and SnoRNA-4, SnoRNA-5, alongside four highly downregulated piR-1374439, piR-949425, SnoRNA-6 and SnoRNA-7. Finally, GO enrichment and KEGG pathway of these 10 significantly different expressed piRNAs, miRNAs and SnoRNAs targeting genes highlighted critical roles in oocyte meiosis, *in utero* embryonic development, insulin signaling pathway and Notch signaling pathway between group L and group S, which is closely related to oocyte and follicle development. Strikingly, GO of these targets gene is rich *in* utero embryonic development (*p*-value 0.003, count ~20) and mitochondrial transport (*p*-value 0.001, count ~30), and these target genes with insulin signaling pathway (*p*-value ~0.01, count ~18) and oocyte meiosis pathway (*p*-value 0.02, count ~8), which converged on follicular development and oocyte maturation pathways.

**Conclusion:**

Our findings demonstrate that follicular fluid EVs SncRNAs orchestrate oocyte developmental competence through size-dependent profiles and pathway activation. These results provide novel insights into improving assisted reproductive technologies in swine and offer potential biomarkers for ovarian follicular selection.

## Background

The relationship between follicle size and oocyte maturation and development is a pivotal area of research within reproductive biology. As follicles increase in size, they experience a series of transformations that are intimately linked to the maturation process of the oocyte (1). Typically, smaller follicles signify earlier phases of development, whereas larger follicles are frequently linked to more progressed stages of oocyte maturation, including competence to respond to epidermal growth factor receptor (EGFR) signaling. Research has indicated that follicles within a specific size range are more likely to harbor oocytes that are capable of undergoing fertilization and subsequent embryonic development (2). Larger follicles provide a better environment for oocyte maturation, with improved hormonal balance and nutrition, resulting in oocytes with higher developmental potential. In contrast, very small follicles may produce less mature oocytes with lower chances of successful fertilization (3, 4), which indicates follicular size closely related to oocyte quality. Follicular fluid contains many extracellular vesicles, which appear to be the maturation of oocytes with the growing and differentiating antral follicle. Extracellular vesicles regulate signaling pathways and foster the growth of oocytes (1). They are crucial for cell communication, transferring proteins, lipids, and nucleic acids, and are involved in immune regulation, tissue repair, and tumor progression (5–8). Recent research found that their potential as biomarkers for diseases and as therapeutic agents (9, 10), including recent evidence for involvement in human subfertility such as poly cystic ovary syndrome (PCOS) (11, 12). Further understanding of their biogenesis, function, and application holds great promise for future medicine and assisted reproductive technology (ART) development (13–16).

Proteomic analyses have revealed a diverse array of proteins within follicular fluid extracellular vesicles, many of which are integral to cell signaling, metabolism, and stress response. Certain proteins may contribute to enhancing oocyte quality and fertility (17, 18). Beyond proteins, follicular fluid extracellular vesicles also encompass nucleic acids, including microRNAs. MicroRNAs are small non-coding RNAs that modulate gene expression by targeting complementary sequences in messenger RNAs (19). Research indicates that microRNAs from follicular fluid extracellular vesicles can be transmitted to the oocyte, potentially affecting its development and maturation (20). The influence of extracellular vesicles on antral Follicle development and fertility extends beyond their direct impact on the oocyte (21). Extracellular vesicles may also alter the function of other follicular cells, such as granulosa and theca cells (6, 22). For instance, they could foster angiogenesis in the follicle by encouraging the proliferation and migration of endothelial cells (6, 23). A deeper understanding of extracellular vesicles' role in follicular fluid could be pivotal for diagnosing and treating infertility. For example, examining follicular fluid extracellular vesicles might offer a non-invasive method to evaluate oocyte quality and forecast the success of assisted reproductive technologies. Moreover, adjusting the content or function of extracellular vesicles could present a novel strategy for enhancing fertility (24). In summary, the exploration of extracellular vesicles in follicular fluid is an emerging field brimming with potential to advance our comprehension of antral Follicle development and female fertility. Further research is essential to fully understand the molecular mechanisms by which extracellular vesicles control follicular function and to investigate the clinical applications of this understanding. In humans, dysregulated expression of exosomal SncRNAs in follicular fluid has been linked to impaired oocyte quality in subfertile patients. For instance, reduced miR-29b levels in human follicular fluid (HFF) correlate with diminished granulosa cell viability and altered steroidogenesis in women with endometriosis or PCOS (25). Additionally, piRNA signatures in HFF extracellular vesicles have been proposed as non-invasive biomarkers for predicting oocyte developmental competence in ART (26).

Based on previous research and current knowledge, it is highly probable that extracellular vesicles play a crucial role in the communication and regulation of surrounding cells in the follicular fluid during oocyte maturation (27). These extracellular vesicles are likely crucial for the relationship and regulation of cells in the growing follicle during oocyte development, through their biological materials like SncRNAs (miRNA, piRNA, snoRNAs, tsRNA, and rsRNA). Despite advances in understanding oocyte quality and development, the molecular mechanisms contributing to high developmental competence are still unclear. Therefore, we aimed to study and compare the relative abundance of the expression of extracellular vesicles containing SncRNAs transcripts from the porcine follicular fluid (PFF) of between group large follicles and group small follicles using Pandora sequence methods, which resolve the problem of these modifications hindering reverse transcriptase passage (28), and identify previously undetected modified SncRNAs, mostly tsRNAs, rsRNAs, piRNAs, and small nucleolar RNAs (snoRNAs), which has redefined snoRNAs as multifunctional regulators of both ribosome biology and gametogenesis (29, 30).

## Methods

### Porcine follicular fluid samples collection

**Figure 1:**
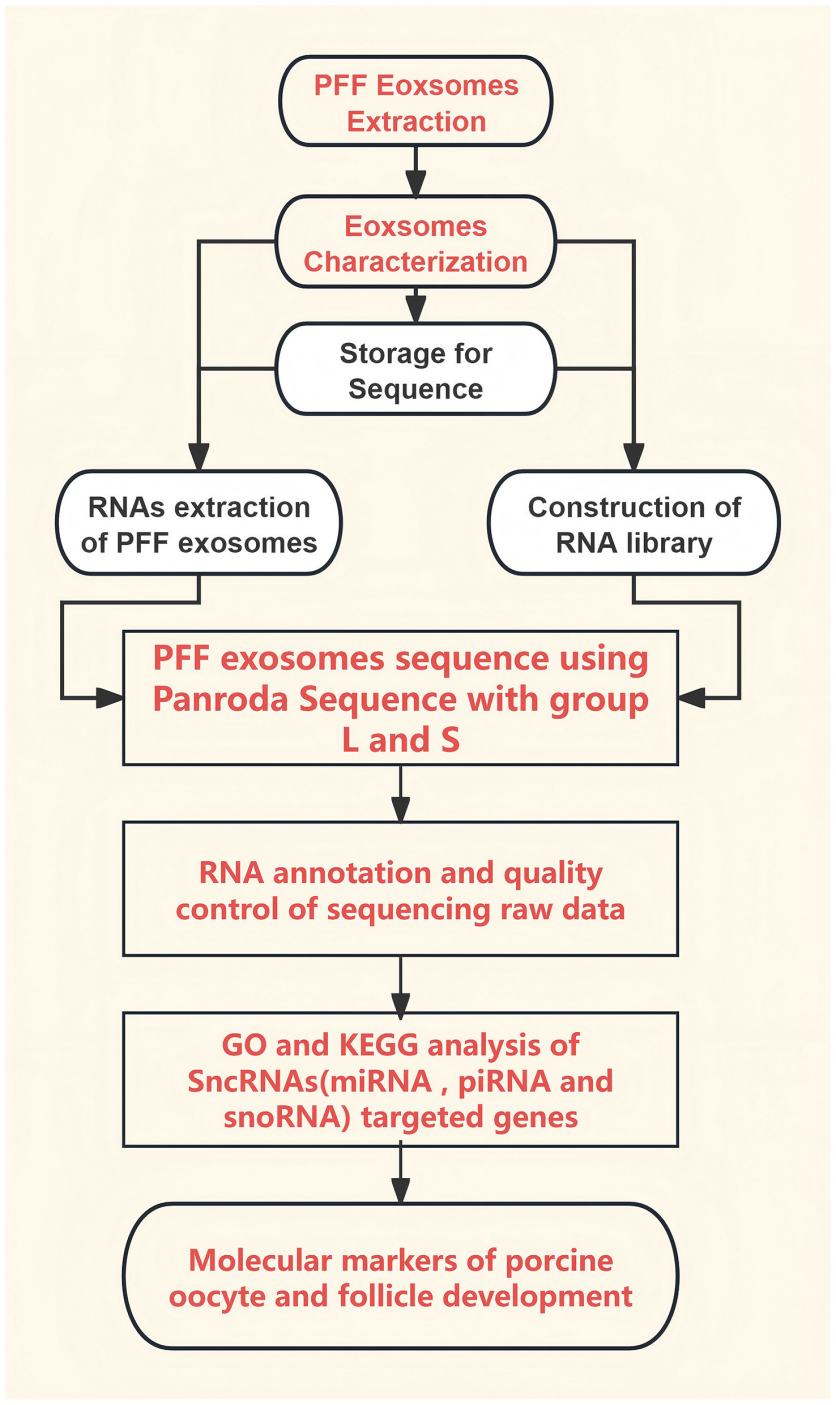
Flowchart of the experiment. A flowchart of the process for the PFF sample collection, identification, Pandora sequence, and SncRNAs analysis between group L and S.

Experimental porcine ovaries were obtained from a local abattoir (QinYangMuYe Limited Responsibility Company, LouDi city, HuNan Province, China) and promptly transferred to our laboratory in a thermos—flask with penicillin and streptomycin within 2 h of slaughter. The experimental outline is described in Figure 1. The ovaries were meticulously washed multiple times with physiological saline solution. Porcine follicular fluid was then extracted by using a 23-gauge needle attached to a 5 ml syringe for small follicles [ < 3 mm (group S)] and a 20-gauge needle attached to a 10 ml syringe for large follicles [>6 mm (group L)] (31). The diameter of each follicle was measured as described previously (1). A total of 300 porcine follicles were aspirated with an average of 0.6 ml of follicular fluid per ovary pair. Subsequently, all follicular fluid samples underwent centrifugation at 1,300 × g for 15 min to eliminate cells, blood, and other debris, and were stored at −80 °C for further analysis.

### EVs purification and characterization

Porcine follicular fluid PFF extracellular vesicles were isolated and characterized following established protocols with minor adjustments (32). Initially, 12 pooled samples (each 15 ml) underwent centrifugation at 1,500 × g for 15 min at 4 °C to remove debris. The resulting supernatant was then transferred to separate 15 ml ultracentrifuge tubes and ultra centrifuged at 16,500 × g for 30 min at 4 °C. Subsequently, the supernatant was filtered through a 0.2 μm syringe filter to collect the EVs-containing medium. Finally, extracellular vesicles were pelleted by ultracentrifugation at 120,000 × g for 70 min at 4 °C. And the supernatant was carefully aspirated, leaving approximately 50 μL of liquid to avoid disturbing the EVs pellet. The pellet was then washed twice to remove contaminating proteins. After the final wash, the supernatant was completely removed, and the purified EV pellet was resuspended in 100 μL sterile PBS and stored at −80 °C until further analysis.

For EVs surface markers analysis, these were quantified using a NanoFCM EX3000 nanoflow cytometer (NanoFCM, Xiamen, China) with 488 and 640 nm laser excitation. Briefly, 20 μL isolated EVs were diluted to 60 μL with sterile PBS, and 30 μL aliquots were incubated with 20 μL fluorescently conjugated primary antibodies (anti-CD63-PE, anti-CD81-FITC; 1:100, System Biosciences) for 30 min at 37 °C in the dark. After labeling, EVs were washed twice by ultracentrifugation [110,000 × g, 70 min, 4 °C, Beckman Coulter SW41Ti rotor (Beckman Coulter, Inc., Brea, CA, USA)]] to remove unbound antibody; supernatants were aspirated, and pellets resuspended in pre-chilled PBS between washes. Following final centrifugation, EVs were resuspended in 50 μL pre-chilled PBS and filtered through a 0.22 μm polyethersulfone (PES) membrane to remove aggregates. Instrument performance was validated with 100 nm fluorescent polystyrene beads before analysis. Samples were serially diluted (1:10–1:100 in PBS to achieve optimal event rates (500–1,000 events/s) and prevent microfluidic channel clogging. Data were acquired using NanoFCM Analysis Software v2.3 (NanoFCM Co., Ltd., Xiamen, China), with ≥10,000 EVs events recorded per sample.

For electron microscopy (EM) analysis, EVs pellets were resuspended in PBS. Samples (5–10 μL each) were added to a copper mesh and allowed to precipitate for 3 min, with excess liquid absorbed using filter paper. Following a rinse with PBS, negative staining with phosphotungstic acid was conducted. The concentration of EVs used for EM preparation is 1.13E+12 (particles/ml) for group S, and 1.85E + 11 (particles/ml) for group L. The samples were air-dried at room temperature for 2 min before imaging (EM operating voltage 80–120 kV). EVs size distribution and concentration were quantified using Nanoparticle Tracking Analysis [NTA; Malvern Panalytical NanoSight NS300 (Malvern Panalytical Ltd., Malvern, UK)]. Resuspended EVs pellets were diluted in sterile PBS (1:500) to achieve 20–100 particles per frame. Three videos of 60-s duration were captured per sample under constant flow (flow rate: 50 μL/min) at 25 °C. Data analysis was performed with NTA Software v3.4 (detection threshold: 5; camera level: 14). NTA measurements from six biological replicates (each with three technical replicates were carried out.

### Construction of RNA library

Total RNA from extracellular vesicles of PFF was extracted using TRIzol Reagent (Invitrogen, Carlsbad, CL, United States). For each sample, 30 μL purified EV pellet (almost 3.3–5.5E + 8 particles) in PBS stored at −80 °C was used for RNA isolation using the miRNeasy Mini Kit according to the manufacturer's protocol. Small RNAs ranging from 15 to 45 nucleotides were selected. Contamination by cell-free RNA is prevented by a two-step workflow involving isolation of EVs prior to RNA extraction, rather than direct RNA extraction from raw bodily fluids. The total RNA extracted from each sample ranged from 3,000 to 12,500 ng as shown in Supplementary Table 1.

The RNA was then incubated in a 50 μl reaction mixture containing 50 mM HEPES (pH 8.0; Gibco, Grand Island, NY, United States) and 75 μM ferrous ammonium sulfate (pH 5.0), 1 mM α-ketoglutaric acid (Sigma-Aldrich; St. Louis, MO, USA), 2 mM sodium ascorbate, 50 mg/L bovine serum albumin (Sigma-Aldrich; St. Louis, MO, USA), 4 μg/ml AlkB (Guangzhou Epibiotek Co., Ltd., Guangzhou, China) with application to remove alkylated RNA modifications such as m1A, m1G, m3C, and m2^2^G, 2,000 U RNase inhibitor, and 200 ng RNA at 37 °C for 30 min. Subsequently, the mixture was added to 500 μL TRIzol reagent to carry out the RNA isolation procedure.

The RNA was further incubated in a 50 μL reaction mixture containing 5 μL 10 × PNK buffer (New England Biolabs, Beverly, MA, United States), 10 mM ATP (New England Biolabs; Beverly, MA, United States), 10 U T4PNK (New England Biolabs, Beverly, MA, United States), and 200 ng RNA at 37 °C for 20 min. The mixture was then added to 500 μl TRIzol reagent to perform the RNA isolation procedure once more. The RNA segment was separated by PAGE, and a 15–45-nucleotide band was selected and recycled. Adapters were obtained from the QIAseq^®^ miRNA Library Kit (QIAGEN, Duesseldorf, Germany) and ligated sequentially. The amplified flow cell was sequenced on the Illumina system by Epibiotek company (Guangzhou Epibiotek Co., Ltd., Guangzhou, China).

### Small RNA annotation and quality control of Pandora sequence data

Small RNA sequences were annotated using the updated software SPORTS 1.1 (successor to SPORTS 1.0), allowing for one mismatch tolerance (SPORTS 1.1 parameter setting: -M1). Reads were sequentially mapped to the following individual non-coding RNA databases: (1) the miRNA database miRBase 21; (2) the genomic tsRNA database GtRNAdb; (3) the mitochondrial tsRNA database mitotsRNAdb; (4) the rsRNA and YRNA databases compiled from the National Center for Biotechnology Information nucleotide and gene database; (5) the piRNA databases, including piRBase and piRNABank; and (6) the non-coding RNAs defined by Ensembl and Rfam 12.3. The tsRNAs were annotated based on both pre-tsRNA and mature tsRNA sequences. Mature tsRNA sequences were derived from the GtRNAdb and mitotRNAdb sequences using the following procedures: (1) predicted introns were removed; (2) a CCA sequence was added to the 3' ends of all tsRNAs; and (3) a G nucleotide was added to the 5′ end of histidine tsRNAs. The tsRNAs were categorized into four types based on the origin of the tsRNA loci: 5′' tsRNA (derived from the 5′ end of pre-/mature tsRNA); 3' tsRNA (derived from the 3′ end of pre-tsRNA); 3′ tsRNA-CCA end (derived from the 3′ end of mature tsRNA); and internal tsRNAs (not derived from 3′ or 5′ loci of tsRNA). For the rsRNA annotation, we mapped the small RNAs to the parent rsRNAs in an ascending order of rsRNA sequence length to ensure a unique annotation of each rsRNA (for example, the rsRNAs mapped to 5.8S rsRNA would not be further mapped to the genomic region overlapped by 5.8S and 45S rsRNAs). Differentially expressed SncRNA analysis was performed using the R package DESeq2.

EVs pellets were resuspended in phosphate-buffered saline (PBS) for subsequent electron microscopy analysis to further verify their characterization according to previously published results (24). The raw sequence data have been deposited in the Genome Sequence Archive (Genomics, Proteomics & Bioinformatics 2021) in National Genomics Data Center (Nucleic Acids Res 2022), China National Center for Bioinformation/Beijing Institute of Genomics, Chinese Academy of Sciences (GSA: CRA017013) that are publicly accessible at https://ngdc.cncb.ac.cn/gsub/submit/gsa/subCRA026922. FastQC software (https://www.bioinformatics.babraham.ac.uk/projects/fastqc/) was used to perform a full evaluation of the sequenced data, including length distribution, quality score, and GC content. The quality control of the raw sequencing data sought to provide a quick impression of whether the data had any problems. This step was necessary to ensure awareness of any problems prior to further analysis according to a previously published method (33, 34). Subsequently, the reads were aligned to a swine reference genome (Sscrofa11.1, January 2017) obtained from the Swine Genome Sequencing Consortium project (https://github.com/Ensembl). Differential gene expression analysis was performed using the DEBseq-counts algorithm, with criteria set at Log_2_FC>1 or FDR < 0.05 for significance.

### GO and KEGG pathway analyses

The hierarchical structure of GO facilitated the organization of mutual control and subordinate relationships into a database. By constructing a functional relationship network, it was possible to identify the functional groups affected by the experiment and the intrinsic subordinate relationships of significant functions. Functional regulation analysis was conducted using significant GO-Terms (*P* < 0.01) from GO-Analysis on differential genes to establish a functional regulatory network. GO classification provides a comprehensive description of gene functions across three main groups: Biological Process (BP), Molecular Function (MF), and Cellular Component. Pathway analysis relies on a gene annotation database to identify significant pathways of differentially expressed genes. The key to successful pathway analysis lies in having a complete database with comprehensive pathway annotations. Those differentially expressed SncRNAs were used for GO or KEGG analysis with 49 piRNAs, 26 miRNAs and 29 snoRNAs. In this study, differentially expressed genes were annotated using the pathway approach based on the KEGG database (https://www.genome.jp/kegg/, release 111.1, September 1, 2024), leading to the identification of pathway terms involving both the differentially expressed genes and their target genes.

Functional annotation was carried out using the Database for Annotation, Visualization and Integrated Discovery (DAVID) (Frederick National Laboratory for Cancer Research, NIH/NCI, Frederick, MD, USA) v6.8 (https://davidbioinformatics.nih.gov/), while pathway analysis utilized annotation data from KEGG (http://www.genome.jp/kegg/) and Cytoscape (https://cytoscape.org/) software. For differentially expressed miRNAs (Threshold: log_2_FC > 2 and *P*-value < 0.05, which is the same as piRNAs and snoRNAs), we predicted their potential target genes using three databases: miRecords, miRTarBase, and TarBase (https://jingege.shinyapps.io/jingle_molecular/). For potential target genes of miRNAs, we conducted GO and KEGG functional enrichment analysis using the DAVID website (https://davidbioinformatics.nih.gov/summary_new.jsp). GO and KEGG analysis were performed using clusterProfiler R package (v3.6.0), with enrichment significance evaluated by a hypergeometric test and multiple testing correction conducted using the Benjamini–Hochberg method.

## Statistical analysis

The back-spliced junction reads and the linear mapped reads were merged and normalized to reads per million mapped reads (RPKM) for quantifying SnRNAs expression levels. Student's *t*-test was employed to analyze the differences in SnRNAs expression profiles among group L and S, with statistical significance set at *P* < 0.05. The R package used this time is DESeq2, and the conditions for screening significant differential genes are: Fold change > 2 and *P* < 0.05.

## Results

### EVs vesicles isolation and identification

**Figure 2:**
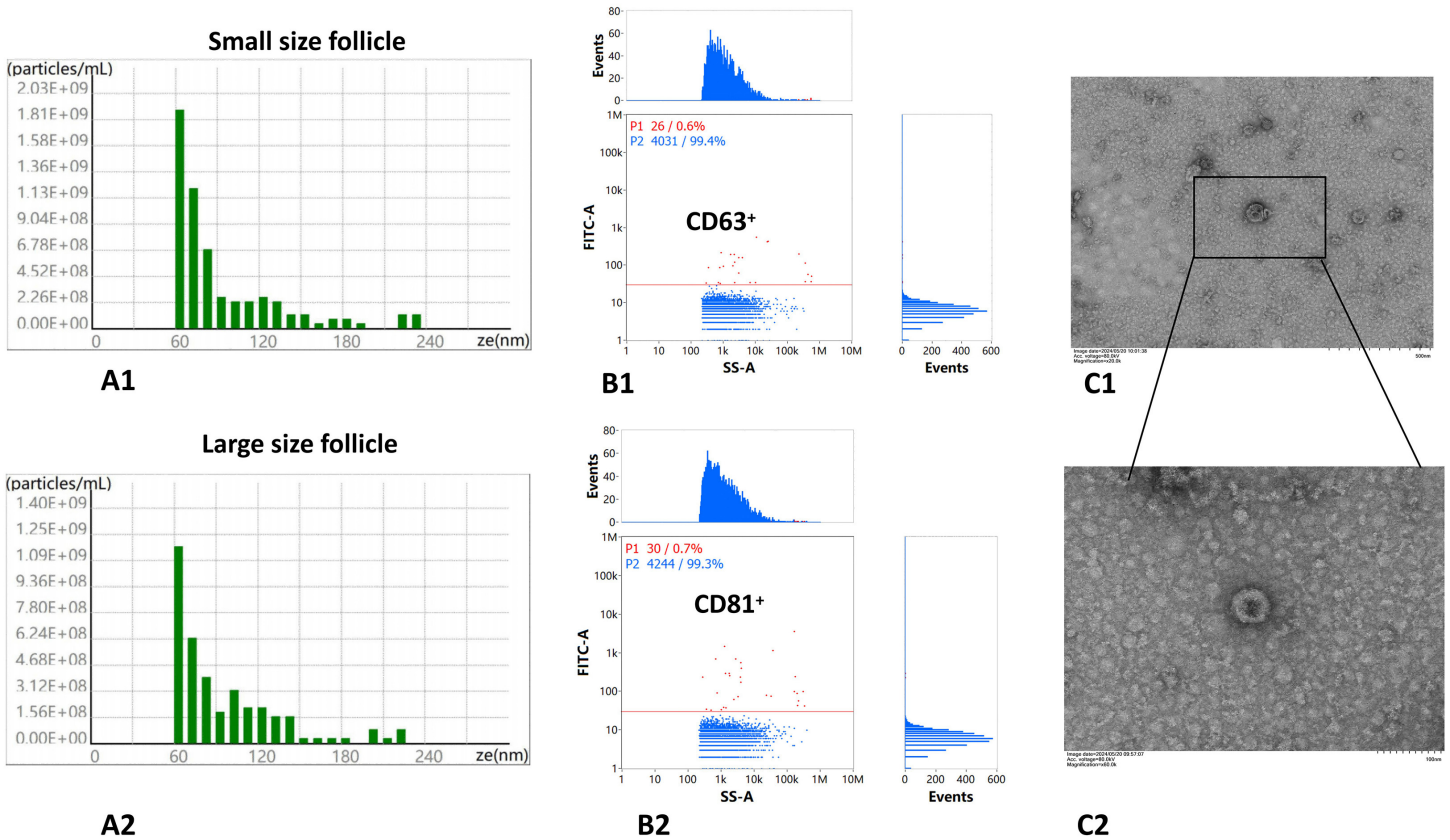
PFF EVs confirmation using transmission electron microscopy (TEM). **(A1)** Measure of extracellular vesicles particle size (nm) and concentration (particles/ml) using nanoparticle tracking analysis (NTA) from group small follicles (S). **(A2)** Measure of extracellular vesicles particle size (nm) and concentration (particles/ml) using nanoparticle tracking analysis (NTA) from group large follicles (L). **(B1)** Marker of extracellular vesicles membrane protein expression is positive for CD63, which 99.4% of checking vesicles are positive. **(B2)** Marker of extracellular vesicles membrane protein expression is positive for CD 81 using Nanoflow cytometry, which 99.3% of checking vesicles are positive. **(C1)** TEM of extracellular vesicles from PFF follicles fluid imaged using 100 nm size microscope rulers. **(C2)** TEM of extracellular vesicles from PFF follicles fluid imaged using 500 nm microscope rulers.

These PFF extracellular vesicles were successfully separated and further verified according to the above methods. Measure of these extracellular vesicles particle size (nm) and concentration (particles/ml) is using nanoparticle tracking analysis (NTA) from group L and S (Figures 2A1, A2). NTA confirmed EVs-sized particles (mode: 102.6 ± 8.4 nm for Group L; 98.3 ± 7.1 nm for Group S; Figures 2A1, A2), with concentrations of (2.1 ± 0.3) × 10^10^ particles/ml and (1.8 ± 0.2) × 10^10^ particles/ml, respectively. These findings align with established EVs size ranges (5). Detection CD 63 protein expression of extracellular vesicles surface captured onto anti-CD63-coated beads (Figure 2B1), and CD 81 protein expression of extracellular vesicles surface captured onto anti-CD81-coated beads is using Flow cytometry analysis (Figure 2B2). These extracellular vesicles were also analyzed using transmission electron microscopy (JEM-1200EX) with an operating voltage of 80–120 kV (Figures 2C1, C2).

### The SncRNAs landscape of PFF extracellular vesicles between group L and S

**Figure 3:**
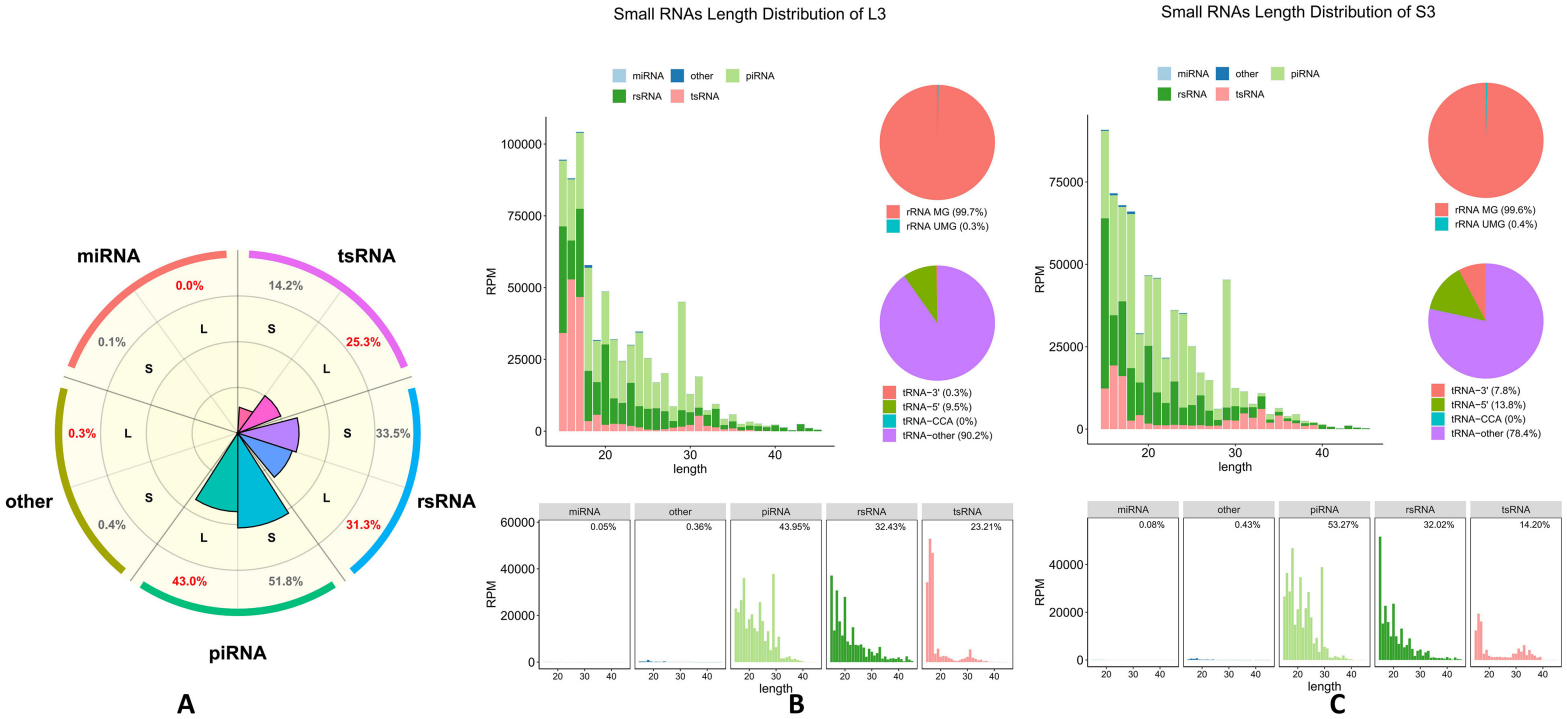
The SncRNAs landscape of PFF extracellular vesicles between group L and S. **(A)** The distribution of distinct SNcRNAs species in large and small follicles. Initially, piRNA makes up 43% in large follicles and 51.8% in small ones. rsRNA constitutes 31.3% and 33.5% in large and small follicles, respectively. tsRNA represents 25.3% in large follicles and drops to 14.2% in small ones. Other RNA types, including miRNA, have a negligible proportion. **(B)** SncRNAs length distribution and classification in large follicles (sample L3). The data reveals that piRNA (43.85%), rsRNA (32.41%), and tsRNA (23.21%) are the most abundant SncRNA classes in large follicles, together accounting for 99% of total reads. Both classes show distinct length preferences: piRNAs and rsRNAs peak at 15–31 nt, while tsRNAs (tRNA-derived small RNAs) are enriched at 15–20 nt, indicating potential roles in gene regulation or stress responses. The proportion of other types of tRNA reached 78.4%, and the proportion of 5' tsRNA was 13.8% in small follicles, and the proportion of 3' tsRNA accounting for 7.8%. **(C)** SncRNAs length distribution and classification in small follicles (sample S3). The data reveals that piRNA (53.27%), rsRNA (32.02%), and tsRNA (14.20%) are the most abundant SncRNA classes in small follicles, together accounting for 99% of total reads. Both classes show distinct length preferences: piRNAs and rsRNAs peak at 15–31 nt, while tsRNAs (tRNA-derived small RNAs) are enriched at 15–18 nt and 30–35 nt.The proportion of other types of tRNA reached 90.2%, and the proportion of 5' tRNA was 9.5%, and the proportion of 3' tRNA accounting for only 0.3%.

Taking into consideration the diverse types of SnRNAs, we compared the proportion of different types of RNA in large and small follicles as shown in Figure 3 (sample L3 compared to sample S3), which other data are shown in Supplementary Figures 1–4 (sample L1–2 compared to sample S1–2). Firstly, piRNA accounts for 43% in the large follicle group, while reaching 51.8% in the small follicle group. The rsRNA, it accounts for 31.3% in the large follicle group and 33.5% in the small follicle group. The tsRNA, it accounts for 25.3% in the large follicle group, but only 14.2% in the small follicle group, and the remaining RNA types (miRNA and other types of RNA) account for a very small proportion (Figure 3A). Accordingly, there appear to exist significant differences in the distribution of different types of RNA in follicles of different sizes.

Next, we conducted a detailed analysis of the subtypes within these two categories of SnRNAs, the rsRNA, and the tsRNA, respectively. Significant differences were found in the proportion of various types of RNA between small and large follicles, with 3′ tsRNA accounting for 7.8% in small follicles and only 0.3% in the large follicle group (Figures 3B, C). For 5′ tsRNA, the proportion was 13.8% in small follicles and 9.5% in the large follicle group (Figures 3B, C). However, in other types of RNA, the proportion reached 90.2% in large follicles but comprised only 78.4% in small follicles (Figures 3B, C). No significant differences were observed in the rsRNA types between the small and large follicle groups. About the length of different SnRNAs types, it shows distinct length preferences: piRNAs and rsRNA peak at 15–31 nt with group L and S (Figures 3B, C), while tsRNAs (tRNA-derived small RNAs) are enriched at 15–20 nt in group L compared to group S with enriched at 15–18 nt and 30–35 nt (Figures 3B, C), indicating potential roles in gene regulation.

### Identification of differentially expressed SncRNAs

**Figure 4:**
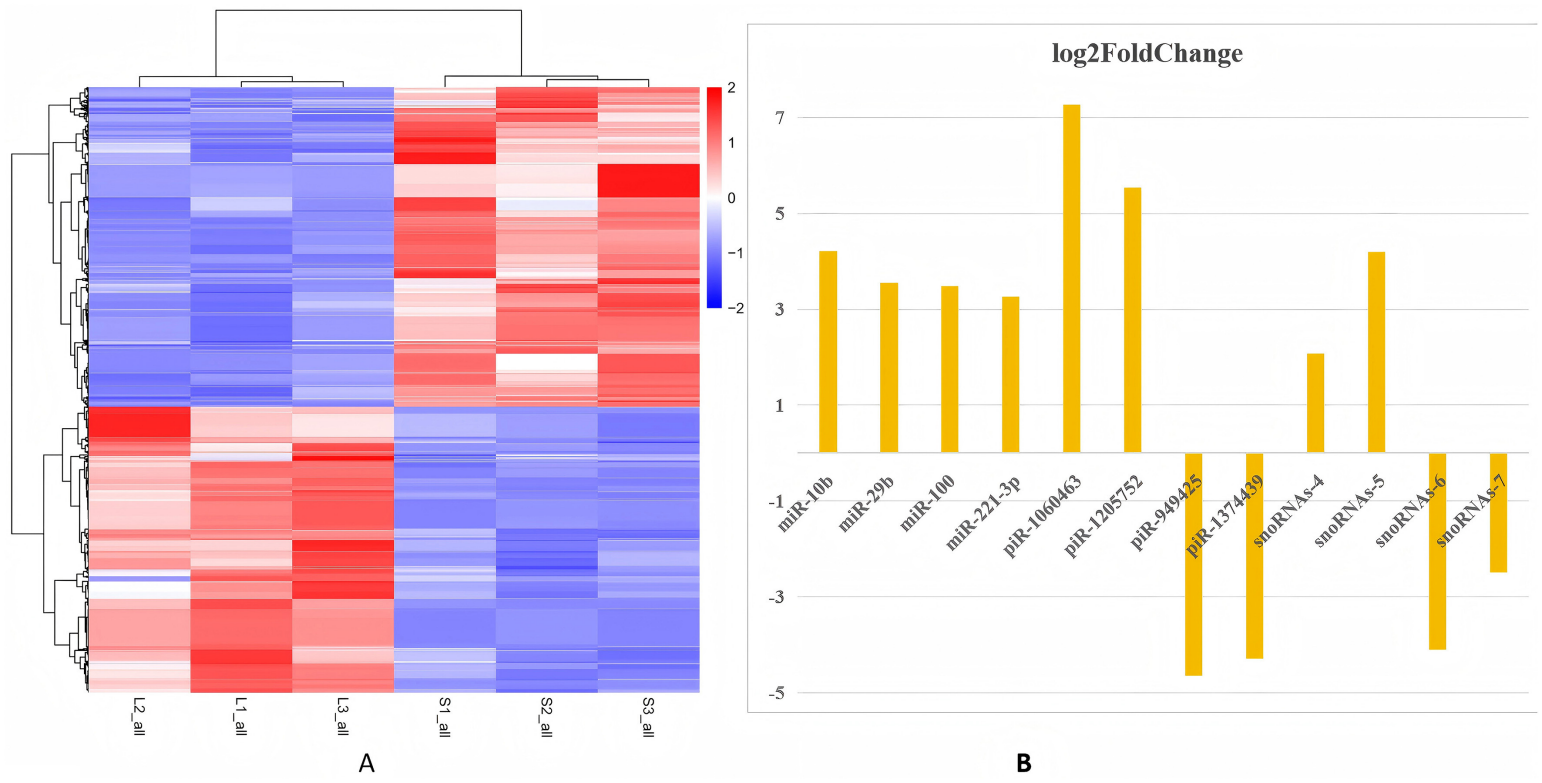
Differential analysis of SncRNA types and the top 12 significantly different expressed genes. **(A)** Heatmap of SncRNAs clustered SncRNA types between groups L and S. Principal expressed global SncRNA profiles shows clear separation between small (< 3 mm, Group S) and large (>6 mm, Group L) follicle extracellular vesicles across three biological replicates per group. **(B)** A horizontal bar chart reveals 12 significant differential expressed analysis of 12 among three different SncRNAs types using the log_2_ fold change value, comparing their expression levels between a follicular development stage (large follicles) and a control stage (small follicles). The *x*-axis represents log_2_FC values, where positive values indicate upregulation and negative values indicate downregulation. The *y*-axis lists individual small RNA species, categorized by type: miRNA (miR-10b, miR-29b, miR-100, miR-221-3p), piRNA (piR-1060463, piR-1205752, piR-949425, piR-1374439), and snoRNA-4, 5, 6, 7. snoRNA-4, GAGAGGGAGAGAACGCGGT; snoRNA-5, GAGAGGGAGAGAACGCG; snoRNA-6, GCCATTGATGATCGT; snoRNA-7, AGGGAGAGAACGCGGT.

**Figure 5:**
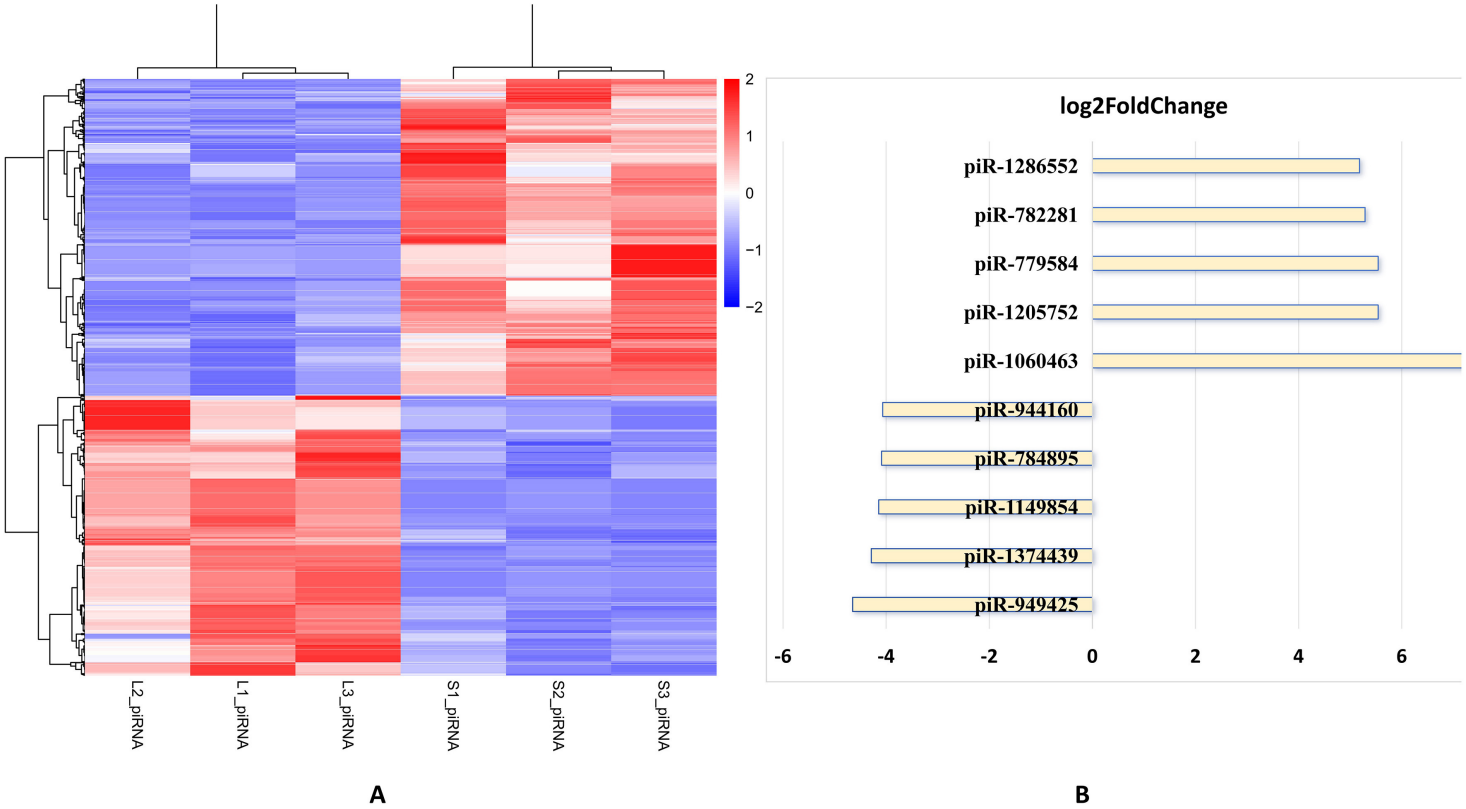
The ten significantly different expressed piRNAs. **(A)** Heatmap of piRNAs clustered genes between group L and S. The expression of piRNA subtypes demonstrates distinct segregation between Group S and Group L. Replicates cluster with minimal dispersion (average Euclidean distance = 1.8). **(B)** Differential expression analysis reveals 5 piRNAs significantly upregulated in group L with piR-1060463 (log_2_FC ~ 7.0), piR-1205752 (log_2_FC ~ 5.0):), piR-1286552 (log_2_FC ~ 6.0), piR-782281 (log_2_FC ~ 5.5) and piR-779584 (log_2_FC ~ 5.0); and 5 downregulated with piR-949425 (log_2_FC ~ −5.0) and piR-1374439 (log_2_FC ~ −3.0), piR-1149854 (log_2_FC ~ −2.5), piR-784895 (log_2_FC ~ −2.0), and piR-944160 (log_2_FC ~ −1.5).

Differentially expressed SncRNA analysis was performed using the R package DESeq 2. The criteria used for screening differentially expressed SncRNAs were log_2_ (Fold Change) > 2 and *P* value < 0.05. For the large follicle group and the small follicle group, three experimental replicates were conducted for each. Subsequent analysis of the sequence data revealed that the distributions of four types of RNA (miRNA, piRNA, rsRNA, and tsRNA) within the three replicates of the large follicle group were clustered together and distinctly differentiated from those of the small follicle group (Figure 4A). This observation suggests that the experimental replicates appear rather homogenous, and the experimental data obtained therefore reliable. The top 12 SncRNAs with significantly differential expression levels in the large follicle group compared to group S, mainly include miRNA (miR-10b, miR-29b, miR-100, miR-221-3p), piRNA (piR-1060463, piR-1205752, piR-949425, piR-1374439), and snoRNA-4, 5, 6, 7 (Figure 4B). Further analysis of the sequence data indicated that the distribution of piRNAs within the three replicates of the large follicle group also clustered together, showing a distinct differentiation from those of the small follicle group (Figure 5A). Concurrently, the top five upregulated piRNAs exhibiting higher differential expression levels in the large follicle group primarily consisted of piR-1060463, piR-1205752, piR-779584, piR-782281, and piR-1286552, whereas the lower expression PiRNAs included piR-949425, piR-1374439, piR-1149854, piR-784895, and piR-944160 (Figure 5B).

**Figure 6:**
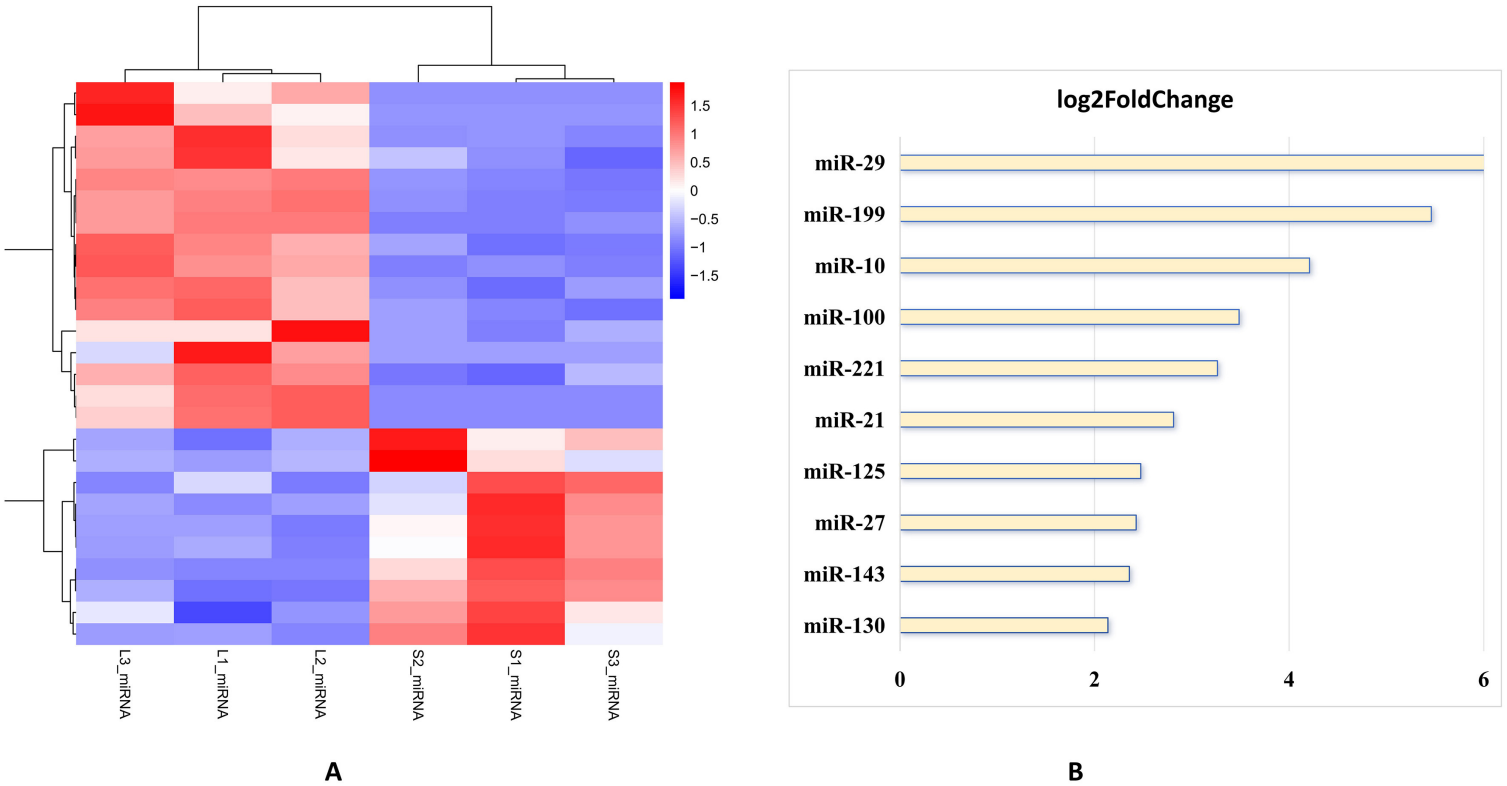
The top ten significantly different expressed miRNAs. **(A)** Heatmap of miRNAs clustered genes between groups L and S, which confirms size-dependent signatures. No inter-group overlap indicates robust biological distinction. **(B)** Most ten significantly expressed miRNAs between group L and S. Top 10 miRNAs upregulated in Group L: miR-29 (log_2_FC ~ 6.0) and miR-199 (log_2_FC ~ 5.0), revealing highly upregulated, and moderately upregulated miRNAs containing miR-10 (log_2_FC ~ 4.0), miR-100 (log_2_FC ~ 3.5), and miR-221 (log_2_FC ~ 3.0), and mildly upregulated miRNAs including miR-21 (log_2_FC ~ 2.5), miR-125 (log_2_FC ~ 2.0), miR-27 (log_2_FC ~ 1.5), miR-143(log_2_FC ~ 1.0), and miR-130 (log_2_FC ~ 0.5), respectively.

**Figure 7:**
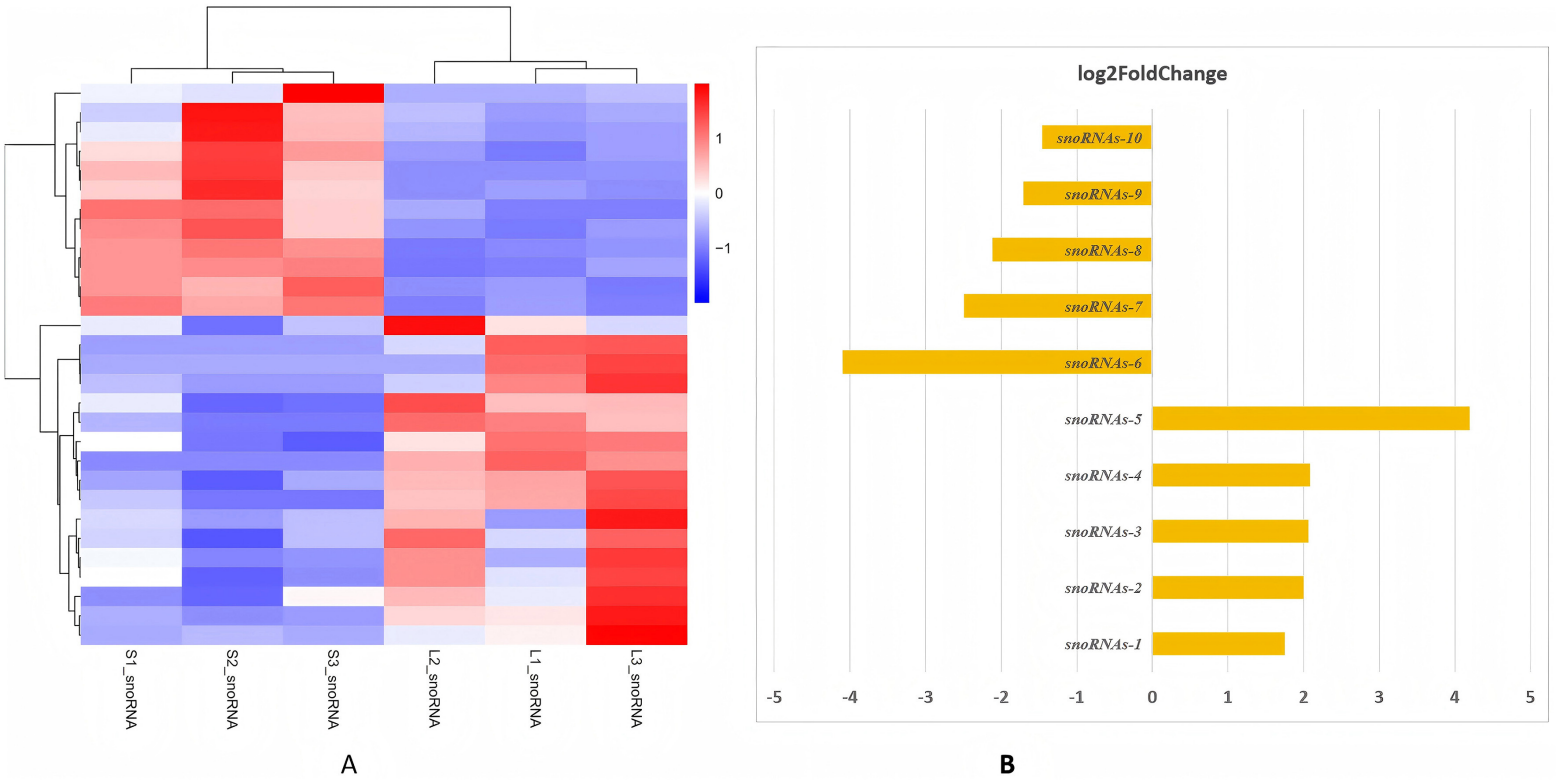
The top ten significantly different expressed snoRNAs. **(A)** Heatmap of snoRNAs clustered genes between groups L and S. The tsRNA subtypes show complete separation between groups. **(B)** The horizontal bar chart illustrates the log_2_fold change (log_2_FC) of 6 small nucleolar RNAs (snoRNAs) with upregulated snoRNA5 (log_2_FC ~ 4.2), snoRNA4 (log_2_FC ~ 2.08), snoRNA3 (log_2_FC ~ 2.06), snoRNA2 (log_2_FC ~ 2.0) and snoRNA5 (log_2_FC~1.75), and with downregulated snoRNA6 (log_2_FC ~ −4.10), snoRNA7 (log_2_FC ~ −2.49), snoRNA8 (log_2_FC ~ −2.12), snoRNA9 (log_2_FC ~ −1.70) and snoRNA10 (log_2_FC ~ −1.45), which compare their expression levels between an antral Follicle development stage (large follicles) and a control stage (small follicles). snoRNA-1, GAGAGGGAGAGAACGC; snoRNA-2, GAGAGGGAGAGAACG; snoRNA-3, GAGAGGGAGAGAACGCGG; snoRNA-8, AGGGAGAGAACGCGG; snoRNA-9, AAAGTGATCGTGGGCT; snoRNA-10, CGGCTCTTGGTGTTGCT.

Subsequent analysis of the sequence data indicated that the distributions of miRNAs within the three replicates of the large follicle group exhibited clustering and were distinctly differentiated from those observed in the small follicle group (Figure 6A). Concurrently, the top 10 miRNAs with significantly higher differential expression levels in the large follicle group primarily comprised miR-130, miR-143, miR-27, miR-125, miR-21, miR-221, miR-100, miR-10, miR-199, and miR-29 (Figure 6B). Further analysis of the sequence data revealed that the distributions of snoRNAs within the three replicates of the large follicle group exhibited clustering, and were clearly differentiated from those observed in the small follicle group (Figure 7A). Concurrently, the 10 snoRNAs with the highest differential expression levels in the large follicle group primarily consisted of snoRNA-1, snoRNA-2, snoRNA-3, snoRNA-4 and snoRNA-5. Additionally, snoRNAs with down expression levels, such as noRNA-6, snoRNA-7, snoRNA-8, snoRNA-9, and snoRNA-10 (Figure 7B).

### GO and KEGG pathway analysis for SnRNAs target gene

**Figure 8:**
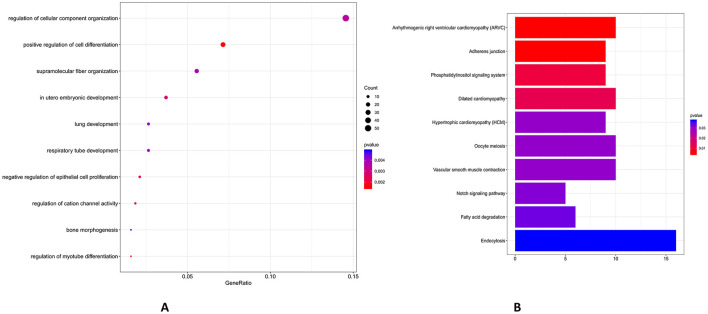
GO enrichment and KEGG analysis of potential target genes of ten significantly different expressed miRNAs. Functional enrichment of miRNA targets identified top biological processes: **(A)** Functional enrichment of miRNA targets identified top biological processes: (1) positive regulation of cell differentiation (*p*-value ~ 0.002, count ~50), (2) supramolecular fiber organization (*p*-value ~ 0.003, count ~30), and (3) *in utero* embryonic development (*p*-value ~ 0.003, count ~20). **(B)** KEGG analysis revealed key pathways: (1) highly significant pathways with adherens junction (*p*-value ~ 0.01, count ~ 12), (2) moderately significant pathways with oocyte meiosis (*p*-value ~ 0.02, count ~8), and (3) developmental and metabolic pathways with Notch signaling pathway (*p*-value ~ 0.03, count ~ 6) and fatty acid degradation (*p*-value ~ 0.03, count ~6). The bar chart is a graphical display of KEGG pathway results. The degree of gene enrichment in the pathway is measured by Rich factor, *P*-value using different colors. Note: the same as following.

Above all, low inter-replicate heterogeneity in SncRNA profiles (Figures 4–7) underscores methodological reproducibility and distinct compositional signatures between follicle size groups, which is the basis for further GO and KEGG pathway analysis. GO functional enrichment analysis indicated that the potential target genes of miRNAs were primarily enriched in cell differentiation, supramolecular fiber organization and *in utero* embryonic development (Figure 8A). KEGG analysis revealed that the potential target genes of differentially expressed miRNAs were predominantly enriched in pathways associated with adherens junction, oocyte meiosis, Notch signaling pathway and fatty acid degradation (Figure 8B).

**Figure 9:**
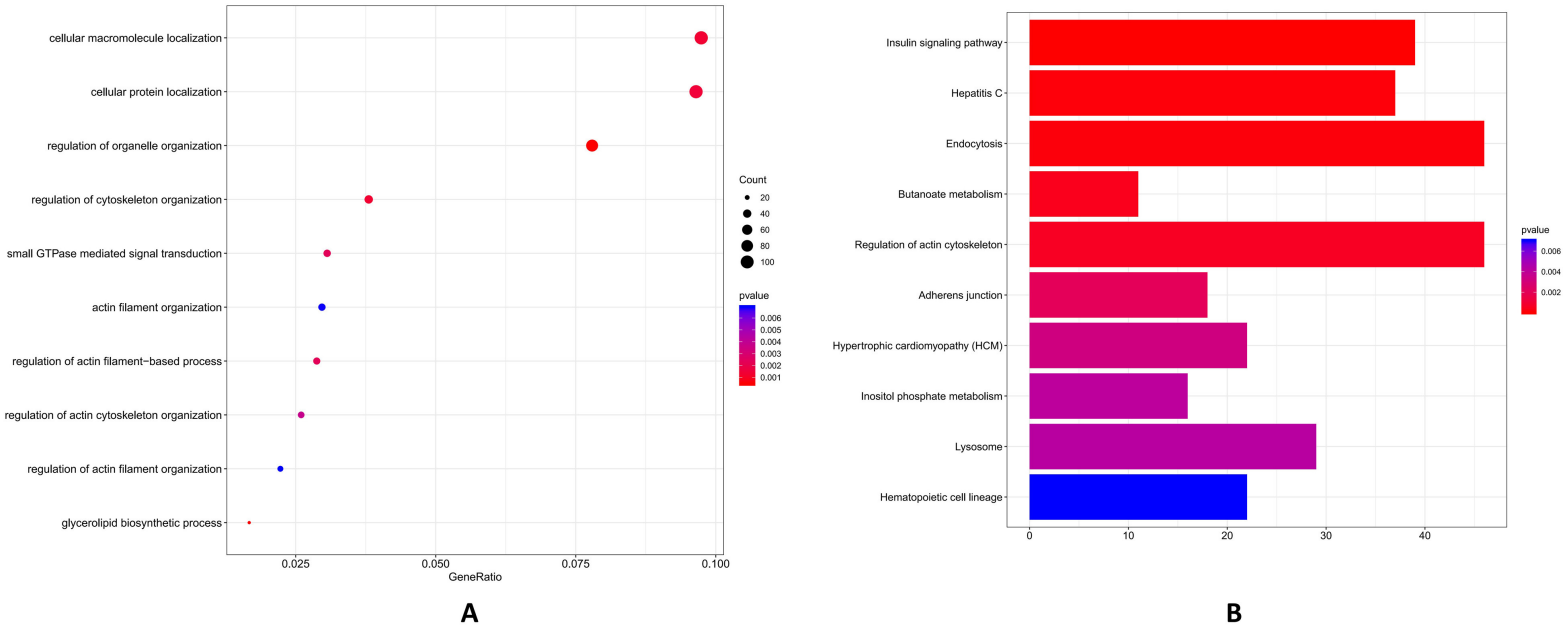
GO enrichment and KEGG analysis of potential target genes of ten significantly different expressed piRNAs. **(A)** piRNA-associated targets showed strongest GO enrichments: (1) cellular macromolecule localization (*p*-value ~0.001, count ~100) and cellular protein localization (*p*-value ~ 0.001, count ~ 100), (2) regulation of organelle organization (*p*-value ~ 0.002, count ~ 80) and regulation of cytoskeleton organization (*p*-value ~ 0.002, count ~ 60), and (3) small GTPase-mediated signal transduction (*p*-value ~ 0.003, count ~ 40) and actin filament organization (*p*-value ~ 0.005, count ~20). **(B)** KEGG pathways included: (1) highly significant pathways with insulin signaling (*p*-value ~ 0.002, count ~40) and Endocytosis (*p*-value ~ 0.002, count ~ 40), (2) moderately significant pathways with regulation of actin cytoskeleton (*p*-value ~ 0.002, count ~ 40), and (3) signaling and adhesion pathways with adherens junction (*p*-value ~ 0.004, count ~ 20) and inositol phosphate metabolism (*p*-value ~ 0.004, count ~ 20).

**Figure 10:**
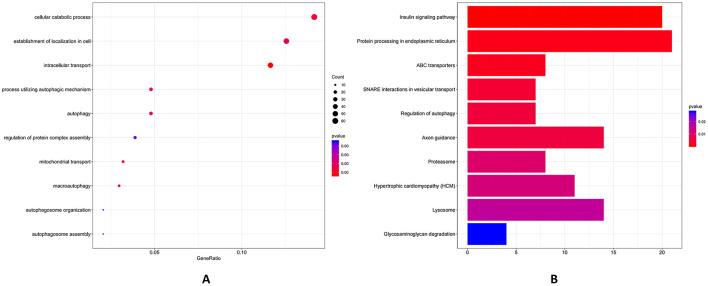
GO enrichment and KEGG analysis of potential target genes of ten significantly different expressed snoRNAs. **(A)** snoRNA target enrichment included critical GO terms: (1) cellular catabolic process (*p*-value ~0.001, count ~ 60), (2) mitochondrial transport (*p*-value ~ 0.001, count ~ 30), and (3) regulation of protein complex assembly (*p*-value ~ 0.004, count ~ 20). **(B)** The top KEGG pathways were: (1) highly significant pathways with insulin signaling pathway (*p*-value ~ 0.01, count ~ 18) and protein processing in endoplasmic reticulum (*p*-value ~ 0.01, count ~ 17), (2) moderately significant pathways with axon guidance (*p*-value ~ 0.01, count ~ 12), and (3) ABC transporters (*p*-value ~ 0.01, count ~ 10) and lysosome (*p*-value ~ 0.02, count ~ 10).

Further, GO functional enrichment analysis indicated that the primary targets of piRNA were predominantly concentrated in cellular macromolecule localization, cellular protein localization, regulation of organelle organization, regulation of cytoskeleton organization, small GTPase mediated signal transduction and actin filament organization (Figure 9A). According to KEGG analysis, the potential target genes of differentially expressed PiRNA between Group L and Group S were predominantly enriched in pathways associated with insulin signaling, endocytosis, regulation of actin cytoskeleton, adherens junction and inositol phosphate metabolism (Figure 9B). GO functional enrichment analysis indicated that the potential target genes of snoRNA were primarily enriched in biological processes, including cellular catabolic process, mitochondrial transport and regulation of protein complex assembly (Figure 10A). KEGG pathway analysis demonstrated that the potential target genes of differentially expressed snoRNA between groups L and S were predominantly associated with pathways such as insulin signaling pathway, protein processing in endoplasmic reticulum, axon guidance, ABC transporters and lysosome (Figure 10B).

## Discussion

It is well established that ovarian follicles create a distinctive microenvironment conducive to oocyte development, facilitating interactions between follicular somatic cells and oocytes (4, 33, 35). Consequently, investigating the constituents of follicular fluid is vital for understanding their role in oocyte maturation mechanisms. Prior research indicates that extracellular vesicles serve as critical carriers for signal transduction between follicular somatic cells and oocytes within follicular fluid (9, 36, 37). Nevertheless, the precise nature of the diverse array of SncRNAs within extracellular vesicles of PFF still remains obscure, therefore it is important to elucidate SncRNAs types and molecular function and pathways related to oocyte and follicle development through using Pandora sequencing technology for finding more according to designed experiment (Figure 1).

The outcomes of the extracellular vesicles size distribution analysis revealed that smaller follicles exhibited a wider size distribution, with a peak at approximately 180 nm, in contrast to larger follicles, which peaked at around 120 nm (Figure 2). The successful isolation and verification of PFF extracellular vesicles were accomplished from PFF, including Nanoparticle Tracking Analysis (NTA), flow cytometry, and Transmission Electron Microscopy (TEM) as shown in Figure 2 according to the utilization of established methodologies (24, 38). In this study, PFF extracellular vesicles were successfully isolated and verified through transmission electron microscopy, yielding results consistent with previous findings (18, 39), which pave a way to further research in SncRNAs of PFF extracellular vesicles.

The results of the SncRNA landscape of PFF extracellular vesicles in large (L) and small (S) follicles reveal notable differences in length distribution and classification. The piRNA (43.85%), rsRNA (32.41%), and tsRNA (23.21%) are the most abundant SncRNA classes in large follicles, together accounting for 99% of total reads as shown in Figure 3A, similar to previous studies indicating tissue-specific RNA distribution patterns (40). Both classes show distinct length preferences: piRNAs and rsRNA peak at 15–31 nt, while tsRNAs (tRNA-derived small RNAs) are enriched at 15–20 nt as shown in Figure 3B, indicating potential roles in gene regulation or stress responses. However, in group S (small size follicle), the piRNA (53.27%) and rsRNA (32.02%) and tsRNA (14.20%) are the most abundant, and the piRNAs and rsRNAs peak at 15–31 nt, while tsRNAs (tRNA-derived small RNAs) are enriched at 15–18 nt and 30–35 nt, as shown in Figures 3A, C, aligning with research highlighting distinct regulatory mechanisms in different biological contexts (41). Analysis of tsRNA subtypes also shows characteristic differences, which the 3' tsRNA has a much higher proportion in small follicles (7.8%) than in large follicles (0.3%) as shown in Figures 3B, C. This is consistent with the idea that different RNA types play crucial roles in follicle development regulation (42). These differences in SncRNAs distribution between small and large follicles may be key to antral follicle development regulation, which contribute uniquely to the biological processes within follicles.

The transcriptomic profiling of SncRNAs revealed a distinct pattern of dysregulation between group L and group S, as visualized in the heatmap (Figure 4A), which indicated that the expression profiles of these regulatory RNAs are strongly associated with the experimental group. The top 12 significantly different expressed genes among three different SncRNAs types (piRNA, miRNA and snoRNA) were shown in Figure 4B, which compared their expression levels between an antral follicle development stage (large follicles) and a control stage (small follicles).

Notably, several miRNAs and piRNAs exhibited significant fold changes (Figure 4B). For instance, miR-10b and miR-29b showed the highest upregulation (log_2_FC ≈ 4.0) and (log_2_FC ~3.0), and piR-120572 showed the highest upregulation (log_2_FC ≈ 7), while piR-949425 was the most downregulated (log_2_FC ≈ −5) as shown in Figure 4B. It has been proved that miR-10b has been linked to cell proliferation and invasion in cancer, but it may promote granulosa cell proliferation to support follicular growth in porcine follicles (43). The published paper also shows that miR-29b is involved in extracellular matrix remodeling, which is essential for follicular expansion and ovulation (44). These findings align with previous studies highlighting the involvement of miR-120572 and piR-949425 in mammalian oocytes and embryos (45). The upregulation of snoRNAs (snoRNA-4 and snoRNA-5) and downregulation of snoRNAs (snoRNA-6 and snoRNA-7) is particularly intriguing, as these molecules are traditionally associated with ribosomal RNA modification. Recent evidence, however, suggests snoRNAs can act as regulators of mRNA stability or translation, implying their potential role in oocyte and follicle development (30). The lack of prior research on snoRNA-4, 5, 6, 7 in this context underscores the novelty of our findings and warrants further investigation into their functional targets.

Furthermore, 10 main significantly different expressed genes among these three SncRNA types (piRNA, miRNA and snoRNA) are chosen by using experimental heatmap between group L compared to group S (Figures 5, 6, 7). Firstly, the distinct expression patterns of piRNAs, like piR-1060463 and piR-1205752, are also notable, which is maintaining genome integrity is crucial for normal oocyte development (41). The marked upregulation of piR-1060463 and piR-1205752 in large follicles aligns with emerging evidence that piRNAs regulate granulosa cell (GC) function. Thus, SncRNAs influence GCs by modulating key biological processes including proliferation, apoptosis, autophagy, cell cycle progression, steroidogenesis, mitochondrial function, inflammatory responses, and aging (11). For instance, (59) identified piR-1060463 as a suppressor of oxidative stress in bovine GCs through mitochondrial retrograde signaling, a mechanism that may explain its association with follicular dominance (46). Conversely, the downregulation of piR-949425 and piR-1374439, suggesting conserved roles in follicular survival. Secondly, among miRNAs, the elevated miR-29b in large follicles reinforces its anti-apoptotic role, as demonstrated by (60) who showed miR-29b inhibits Caspase-3 cleavage in human GCs (47). Similarly, miR-10b upregulation corresponds with its promotion of estradiol synthesis in ovine follicles (43, 48). Notably, the coordinated overexpression of miR-100 and miR-221 (Figure 6B) mirrors their synergistic regulation during oocyte maturation (49), suggesting these miRNAs may collectively enhance follicular competence. The data highlights that miRNAs are key regulators of antral Follicle development, with a subset of highly upregulated miRNAs (e.g., miR-29, miR-199) driving critical processes like extracellular matrix remodeling and cell proliferationas shown in Figure 6, which is similar to the publieshed papers (11, 43, 44). Finally, the results show that snoRNAsare key regulators of antral Follicle development, with a subset of upregulated snoRNAs (e.g., snoRNAs-5, snoRNAs-4) driving ribosome biogenesis to meet the metabolic demands of growing follicles as shown in Figure 7. Downregulated snoRNAs (e.g., snoRNAs-9, snoRNAs-10) likely repress inhibitory pathways, allowing follicular maturation as shown in Figure 7. In conclusion, these findings expand our understanding of SncRNA-mediated mechanisms in reproductive biology, suggesting piRNAs, miRNAs, and snoRNAs may be potential targets for improving oocyte quality and fertility outcomes.

In order to explore the potential target genes of different SncRNAs including piRNAs, miRNAs, and snoRNAs, we performed GO and KEGG analyses of these 10 SncRNAs potential target genes. Firstly, the enrichment in biological processes of 10 miRNA targeted genes mainly focuses on cell differentiation, supramolecular fiber organization, and *in utero* embryonic development (Figure 8A). As known to us, the phosphatidylinositol signaling pathway, significantly enriched in miRNA target genes (Figure 8B), plays a pivotal role in follicle development and oocyte maturation. This pathway mediates critical intracellular processes including calcium homeostasis, membrane trafficking, and cytoskeletal rearrangement—all essential for granulosa cell proliferation, cumulus-oocyte complex expansion, and precise regulation of oocyte meiosis (50). Notably, phosphatidylinositol signaling cross talks with other enriched pathways, which intersects with insulin signaling to modulate nutrient uptake and energy metabolism in oocytes, and with oocyte meiosis to regulate spindle organization and chromosome segregation (51). KEGG analysis showed that pathways like adherens junction, oocyte meiosis, Notch signaling pathway and fatty acid degradation (Figure 8B) were enriched. This indicates that miRNAs may play a direct role in regulating the meiotic process of oocytes, which is a key stage in their development (52). These findings are consistent with previous studies suggesting that miRNAs are important regulators in reproductive processes (31, 53). The results showed that these identified miRNAs (e.g., miR-221, miR-125b) in large-follicle extracellular vesicles that may promote EGFR signaling and suppress inflammation, which is similar to the reports finding that miR-221 targeting estrogen inhibitors, while miR-125b in extracellular vesicles improving oocyte quality by modulating adenosine deaminase RNA (54).

We can show that piRNAs' potential target genes were concentrated in biological processes such as cellular macromolecule localization, cellular protein localization, regulation of organelle organization, regulation of cytoskeleton organization, small GTPase-mediated signal transduction and actin filament organization (Figure 9A). It is also found that piRNAs may regulate GTPase signaling and actin filament organization pathways (e.g., Rho, Ras), which control granulosa cell proliferation and follicular growth, which is reported that dysregulation of these pathways is linked to ovarian disorders (1, 22, 45). KEGG analysis showed enrichments in pathways like insulin signaling, endocytosis, regulation of actin cytoskeleton, adherens junction and inositol phosphate metabolism (Figure 9B). The insulin signaling pathway has previously been reported to be involved in oocyte maturation and ovulation (55). piRNAs may regulate genes in this pathway to control these processes. The regulation of the cytoskeleton, which is also influenced by piRNAs, is necessary for oocyte shape maintenance and meiotic division. The results show that piR-1205752 is upregulated in group L, which is enriched in insulin signaling (Figure 9B), which cross-talks with EGFR to promote cumulus expansion. Finally, GO analysis of snoRNAs' potential target genes indicated enrichments in cellular catabolic process, mitochondrial transport and regulation of protein complex assembly (Figure 10A). Cellular metabolism provides the energy and building blocks required for oocyte growth, and intracellular transport and mitochondrial transport are essential for the proper distribution of organelles and energy generating mitochondria within the oocyte (56). KEGG analysis showed associations with pathways such as insulin signaling pathway, protein processing in endoplasmic reticulum, axon guidance, ABC transporters and lysosome (Figure 10B). Similar to piRNAs, snoRNAs may also regulate oocyte development through the insulin signaling pathway, which is also found in this result (57, 58).

Taken together, the results suggest that there is a complex gene interaction network among miRNAs, piRNAs, and snoRNAs in follicular development. For example, piR-1060463 and miR-29b are differentially expressed and linked to signal transduction pathways, and their specific functions in granulosa cell or oocyte biology require further validation. Our findings raise intriguing possibilities for therapeutic interventions. For example, supplementing *in vitro* maturation (IVM) cultures with large follicle-derived extracellular vesicles rich in miR-29b or piR-1060463 could potentially enhance oocyte meiotic competence by activating insulin signaling pathway or mitochondrial function, as observed in porcine models.

## Conclusions

In conclusion, our study provides insights into the gene regulatory mechanisms of miRNAs, piRNAs, and snoRNAs in oocyte development. The complex interaction network of these SncRNAs and their target genes may play a crucial role in oocyte development. Future research should focus on further elucidating the detailed molecular mechanisms of these regulatory networks.

## Data Availability

The raw sequence data have been deposited in the Genome Sequence Archive (Genomics, Proteomics & Bioinformatics 2021) in National Genomics Data Center (Nucleic Acids Res 2022), China National Center for Bioinformation/Beijing Institute of Genomics, Chinese Academy of Sciences (GSA: CRA017013) that are publicly accessible at https://download.cncb.ac.cn/gsa5/CRA017013/.
