# Supplementary material for: EV-derived small non-coding RNAs from porcine follicular fluid regulate follicle development using Pandora sequence
Source: Front Vet Sci. 2026 Mar 17;13:1754433. doi: 10.3389/fvets.2026.1754433 (PMC13035755; doi:10.3389/fvets.2026.1754433)
Supplement: Supplementary file 1 [file Table_1.docx]

| number | Sample name | Sample number | Dosage（ng/μL） | Volume（μL） | Total weight（ng） | Total Quality Inspection | Quality inspection conclusion |
| --- | --- | --- | --- | --- | --- | --- | --- |
|  | S1 | BGBN242393wPDL | 1380.00 | 9.0 | 12420 | Qualified | Qualified |
|  | S2 | BGBN242394wPDL | 1320.00 | 9.0 | 11880 | Qualified | Qualified |
|  | S3 | BGBN242395wPDL | 1172.00 | 9.0 | 10548 | Qualified | Qualified |
|  | L1 | BGBN242399wPDL | 612.00 | 9.0 | 5508 | Qualified | Qualified |
|  | L2 | BGBN242400wPDL | 484.00 | 9.0 | 4356 | Qualified | Qualified |
|  | L3 | BGBN242401wPDL | 352.00 | 9.0 | 3168 | Qualified | Qualified |

Table S1 RNA integrity, yield, and quality assessment extracted from PFF EVs
