# Supplementary figures and images for: EV-derived small non-coding RNAs from porcine follicular fluid regulate follicle development using Pandora sequence
Source: Front Vet Sci. 2026 Mar 17;13:1754433. doi: 10.3389/fvets.2026.1754433 (PMC13035755; doi:10.3389/fvets.2026.1754433)

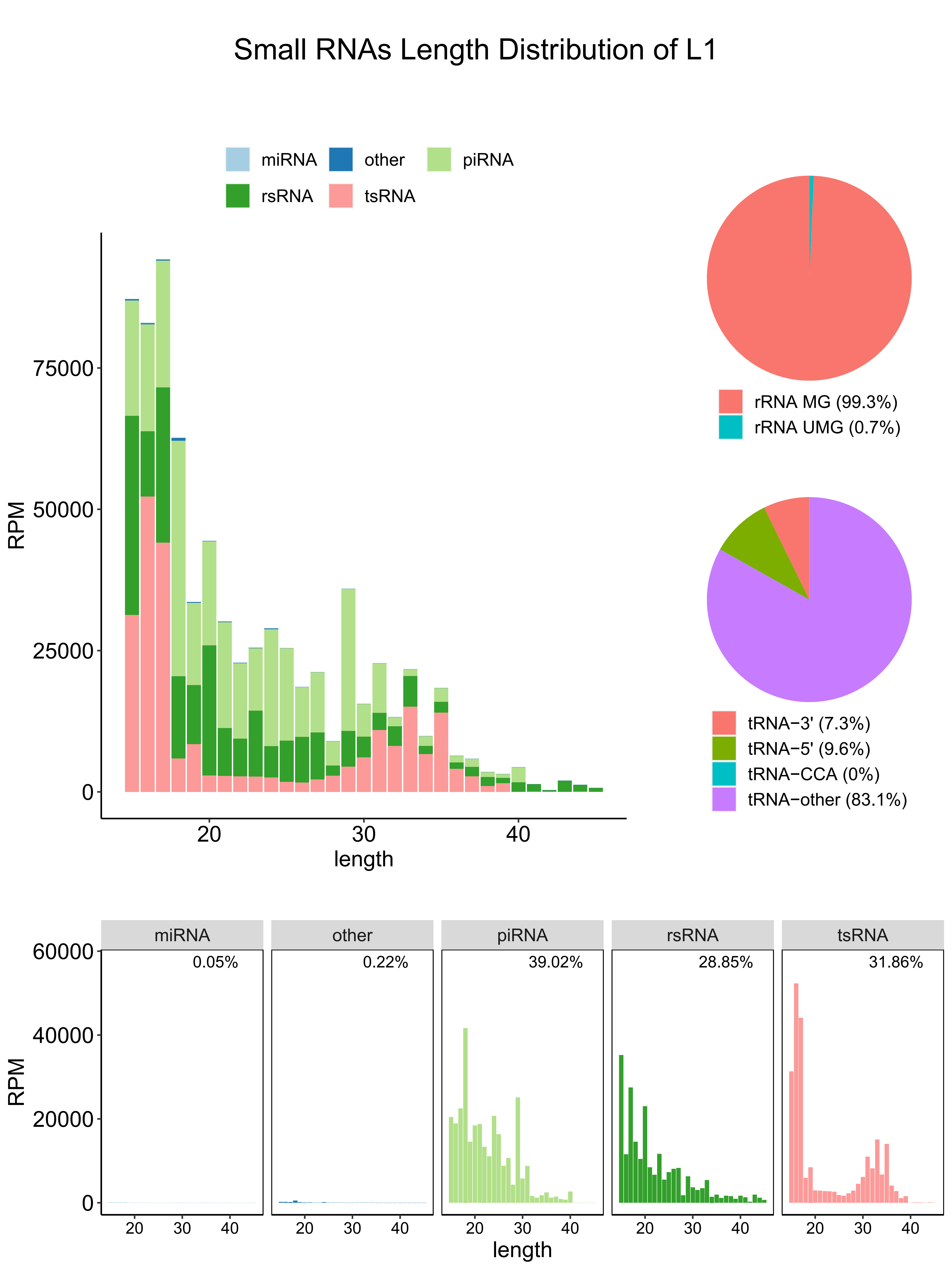

Supplement: Supplementary file 2 [file Image_1.png]

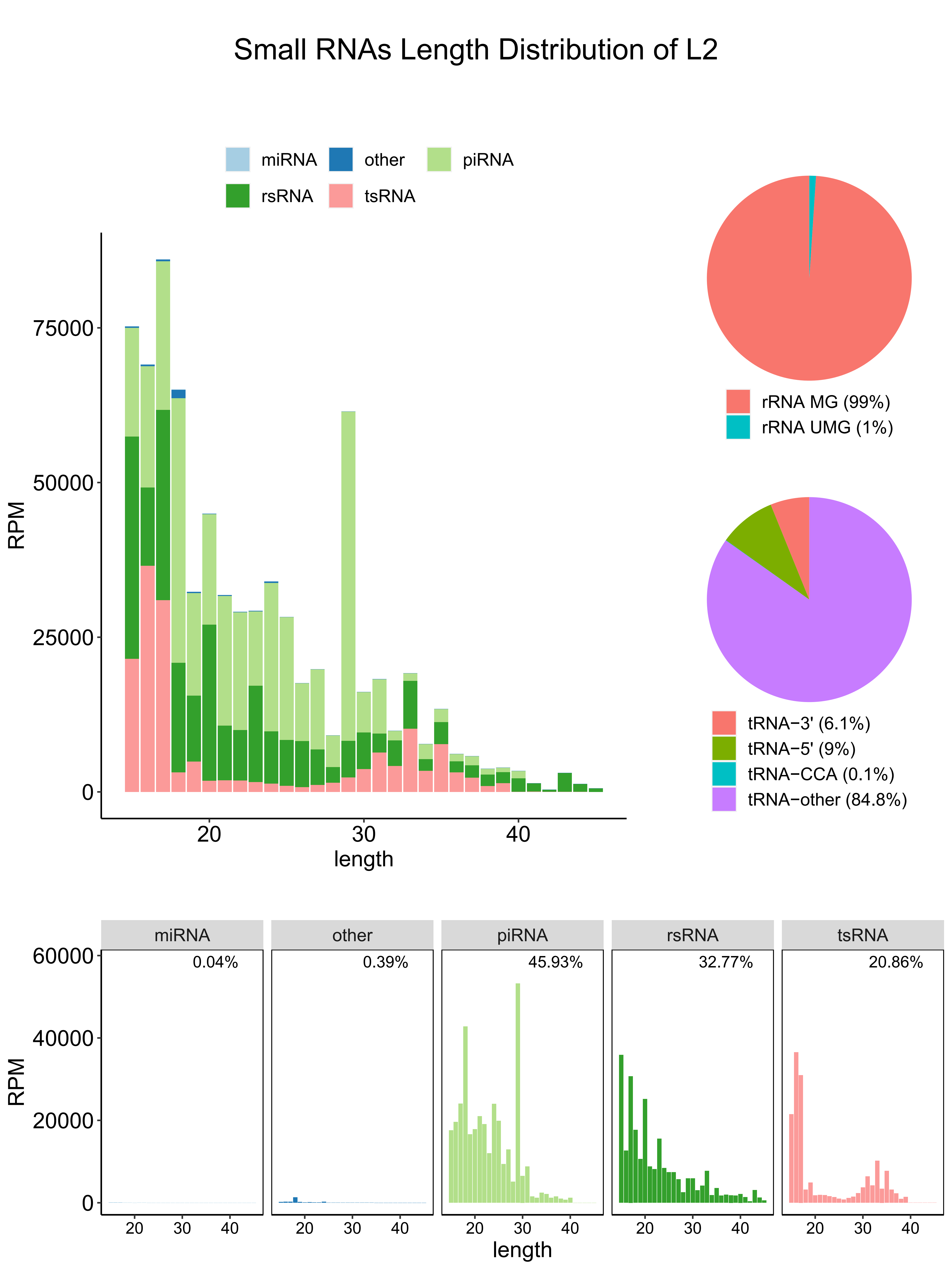

Supplement: Supplementary file 3 [file Image_2.png]

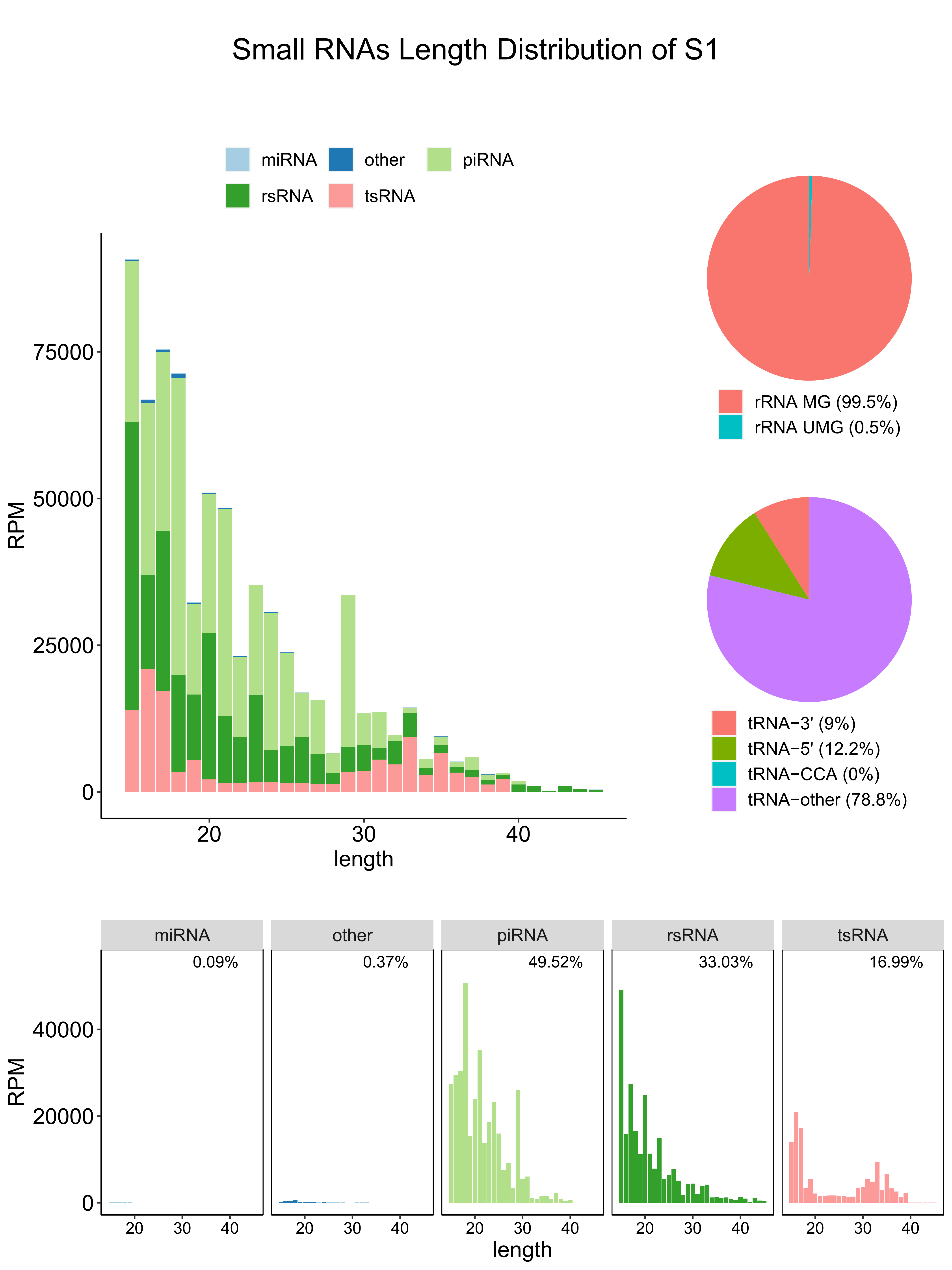

Supplement: Supplementary file 4 [file Image_3.png]

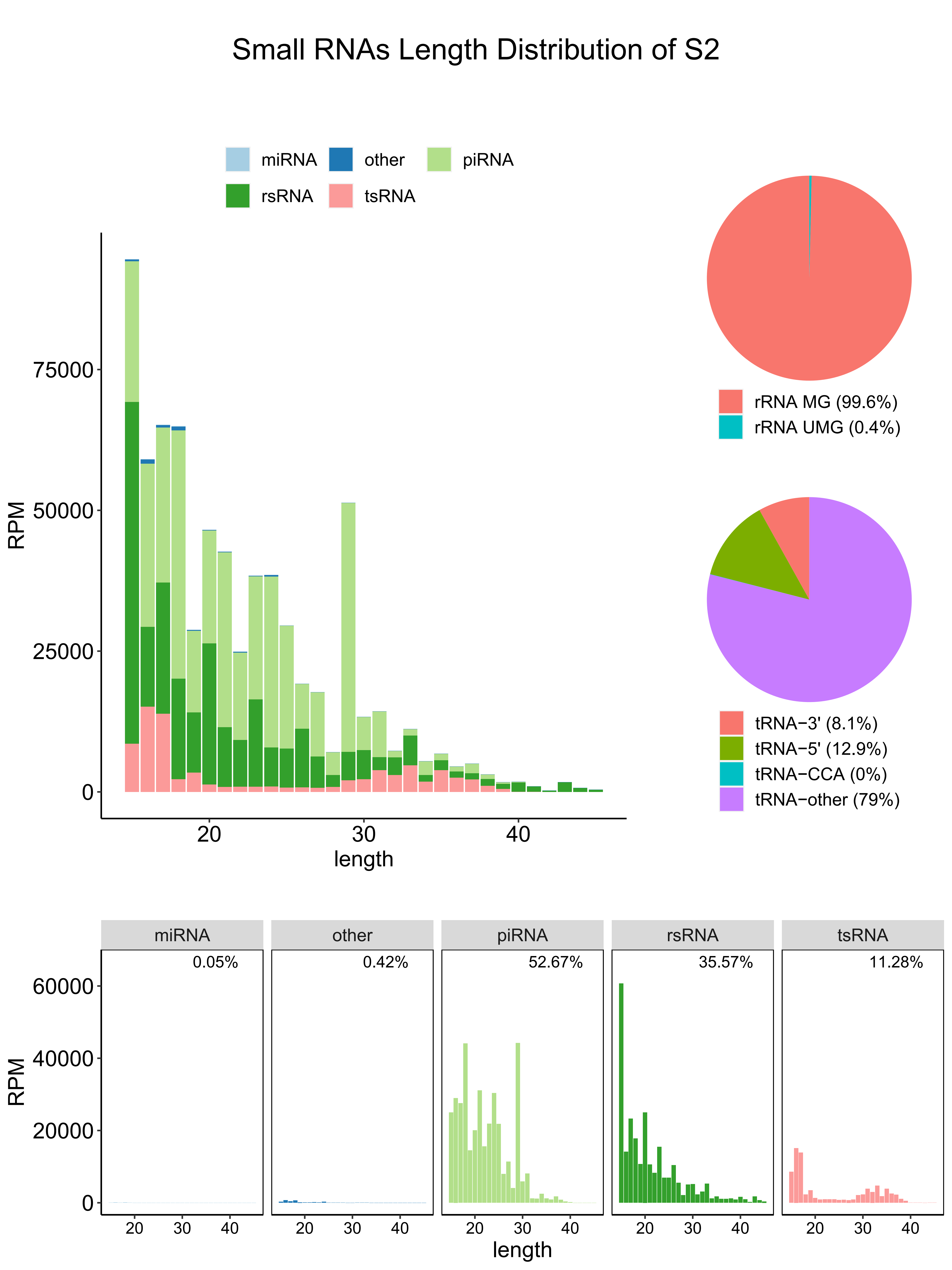

Supplement: Supplementary file 5 [file Image_4.png]
